# Formulation Design of Fast Disintegrating Tablets Using Disintegrant Blends

**DOI:** 10.4103/0250-474X.62244

**Published:** 2010

**Authors:** S. B. Shirsand, Sarasija Suresh, P. V. Swamy, M. S. Para, D. Nagendra Kumar

**Affiliations:** * Department of Pharmaceutical Technology, H. K. E. Society's College of Pharmacy, Sedam Road, Gulbarga-585 105, India; 1 Department of Pharmaceutics, Al-Ameen College of Pharmacy, Hosur Road, Bangalore-560 027, India; 2 S. V. E. T.'s College of Pharmacy, Humnabad-585 330, India

**Keywords:** Cros-carmellose sodium, crospovidone, fast disintegrating tablets, Prochlorperazine maleate

## Abstract

In the present work, fast disintegrating tablets of prochlorperazine maleate were designed with a view to enhance patient compliance by direct compression method. In this method, crospovidone (up to 3% w/w) and croscarmellose sodium (up to 5% w/w) in combination were used as superdisintegrants. Since disintegrants complement each other, accelerating the disintegration process when used together. Estimation of prochlorperazine maleate in the prepared tablet formulations was carried out by extracting the drug with methanol and measuring the absorbance at 254.5nm. The prepared formulations were further evaluated for hardness, friability, drug content uniformity, *in vitro* dispersion time, wetting time and water absorption ratio. Based on *in vitro* dispersion time (approximately 12 s), one promising formulation was tested for *in vitro* drug release pattern in phosphate buffer pH 6.8 and short-term stability (at 40°/70% RH for 3 mo), drug-excipient interaction (IR spectroscopy) were studied. Among the formulations tested, formulation DCPC_4_ containing 5% w/w of croscarmellose sodium and 3% w/w of crospovidone as superdisintegrant emerged as the overall best (t_50%_ 7.0 min) based on drug release characteristics in pH 6.8 phosphate buffer compared to commercial conventional tablet formulation (t_50%_ 17.4 min). Short-term stability studies on the promising formulation indicated that there were no significant changes in drug content and *in vitro* dispersion time (p<0.05).

Many patients express difficult to swallow tablets and hard gelatin capsules and thus does not comply with prescription, which results in high incidence of non-compliance and ineffective therapy. Recent advances in novel drug delivery systems (NDDS) aim to enhance safety and efficacy of drug molecules by formulating a convenient dosage form for administration and to achieve better patient compliance. One such approach is fast dissolving tablets (FDT)[1–4]. Prochlorperazine maleate (PCZM) is a phenothiazine antipsychotic and widely used in prevention and treatment of nausea, vomiting including that associated with migraine or drug-induced emesis[5]. The concept of formulating fast dissolving tablets containing prochlorperazine maleate offers a suitable and practical approach in serving desired objective of faster disintegration and dissolution characteristics with increased bioavailability by simple and cost effective direct compression technique.

PCZM maleate was a gift sample from Mehta Pharmaceuticals, Mumbai, India. Cros-carmellose sodium (CCS) and Crospovidone (CP) were gift samples from Wockhardt Research Centre, Aurangabad. All the other ingredients were of analytical grade.

FDTs of PCZM were prepared by direct compression[6] according to the formulae given in Table 1. All the ingredients were passed through # 60 mesh separately. Then the ingredients were weighed and mixed in geometrical order and compressed into tablets of 150 mg using 8 mm round flat punches on 10-station rotary tablet machine (Clit). A batch of 60 tablets was prepared for all the designed formulations.

**TABLE 1:** COMPOSITION OF DIFFERENT BATCHES OF FAST DISINTEGRATING TABLETS OF PROCHLORPERAZINE MALEATE

Ingredients*	Formulation code				
	
	DC_0_	DCPC_1_	DCPC_2_	DCPC_3_	DCPC_4_
Prochlorperazine Maleate	5.0	5.0	5.0	5.0	5.0
Crospovidone	--	1.5	1.5	1.5	4.5
Croscarmellose sodium	--	1.5	3.0	4.5	7.5
Microcrystalline cellulose (MCC)	--	--	30	30	30
Aspartame	3.0	3.0	3.0	3.0	3.0
Sodium stearyl fumarate	1.5	1.5	1.5	1.5	1.5
Talc	3.0	3.0	3.0	3.0	3.0
Pineapple flavor	1.5	1.5	1.5	1.5	1.5
Mannitol (Pearlitol SD 200)	136	133	101.5	100	94
Total	150	150	150	150	150

* All the quantities expressed are in mg/tablet

Twenty tablets were selected at random and weighed individually. The individual weights were compared with the average weight for determination of weight variation[7]. Hardness and friability of the tablets were determined by using Monsanto hardness tester and Roche friabilator, respectively. For content uniformity test, ten tablets were weighed and powdered. The powder equivalent to 5 mg of PCZM was extracted into methanol and liquid was filtered (Whatmann No. 1 filter paper). The PCZM content in the filtrate was determined by measuring the absorbance at 254.5 nm after appropriate dilution with methanol. The drug content was determined using the standard calibration curve. The mean percent drug content was calculated as an average of three determinations[8]. For determination of wetting time and water absorption ratio[9], a piece of tissue paper folded twice was placed in a small petridish (internal diameter of 5 cm) containing 6 ml of water. A tablet was placed on the paper and the time required for complete wetting was measured. The wetted tablet was then weighed. Water absorption ratio ‘R’ was calculated using the equation: R=100 (W_a_−W_b_)/W_b_; where W_a_ is weight of tablet after water absorption and W_b_ is weight of tablet before water absorption. For determination of *in vitro* dispersion time, one tablet was placed in a beaker containing 10 ml of pH 6.8 phosphate buffer at 37±0.5° and the time required for complete dispersion was determined[10]. IR spectra of PCZM and its formulations were obtained by KBr pellet method using Perkin-Elmer FTIR series (Model 1615) spectrophotometer in order to rule out drug-carrier interactions.

*In vitro* dissolution of PCZM fast disintegrating tablets was studied in USP XXIII type-II dissolution apparatus (Electrolab, Model-TDT 06N) employing a paddle stirrer at 50 rpm using 900 ml of pH 6.8 phosphate buffer at 37±0.5° as dissolution medium[11]. One tablet was used in each test. Aliquots of dissolution medium (5 ml) were withdrawn at specified intervals of time and analyzed for drug content by measuring the absorbance at 255.5 nm. The volume withdrawn at each time interval was replaced with fresh quantity of dissolution medium. Cumulative percent of PCZM released was calculated and plotted against time.

Short-term stability studies on the promising formulations (DCPC_3_ and DCPC_4_) were carried out by storing the tablets in an amber coloured rubber stoppered vial at 40°/75% RH over a period of 3 mo. At intervals of 1 mo, the tablets were visually examined for any physical changes, changes in drug content and *in vitro* dispersion time.

Fast disintegrating tablets of prochlorperazine maleate were prepared by direct compression method using blends of crospovidone and croscarmellose sodium as super-disintegrants in different ratios. Directly compressible mannitol (Pearlitol SD 200) was used as a diluent to enhance mouth-feel. A total of four formulations and a control formulation DC_0_ (without super-disintegrant) were designed. As the blends were free flowing (angle of repose <30°, and Carr's index <15%) tablets obtained were of uniform weight (due to uniform die fill), with acceptable variation as per IP specification i.e., below 7.5%. Drug content was found to be in the range of 95 to 99%, which is within acceptable limits. Hardness of the tablets was found to be in the range of 2.5-2.6 kg/cm^2^. Friability below 1% was an indication of good mechanical resistance of the tablets. Water absorption ratio and wetting time, which are important criteria for understanding the capacity of disintegrants to swell in presence of little amount of water were found to be in the range of 60-76% and 15-51 s, respectively. Among all the designed formulations, one formulation, viz., DCPC_4_ was found to be promising and displayed an *in vitro* dispersion time of 12 s, which facilitates its faster dispersion in the mouth.

Overall, the formulation DCPC_4_ containing 3% w/w of crospovidone and 5% w/w of croscarmellose sodium was found to be promising and has shown an *in vitro* dispersion time of 12 s, wetting time of 15 s and water absorption ratio of 76% when compared to control formulation (DC_0_) which shows 191 s, 185 s and 49% values, respectively for the above parameters (Table 2).

**TABLE 2:** EVALUATION OF FAST DISINTEGRATING TABLETS OF PROCHLORPERAZINE MALEATE

Parameters	Formulation code				
	
	DC_0_	DCPC_1_	DCPC_2_	DCPC_3_	DCPC_4_
Hardness*±SD (kg/cm^2^)	2.53±0.152	2.56±0.152	2.6±0.20	2.5±0.152	2.59±0.02
Thickness (mm)	2.19	2.24	2.29	2.26	2.21
Friability (%)	0.82	0.75	0.84	0.74	0.8
Percent drug content±SD*	97.74±0.62	97.76±0.72	95.68±0.592	99.03±0.78	97.77±0.62
*In vitro* dispersion time±SD* (sec)	185.86±5.79	48.64±0.90	37.03±1.51	26.74±2.25	12.52±0.98
Wetting time±SD* (sec)	191.88±2.94	51.91±2.61	40.52±1.70	30.65±1.80	15.45±0.93
Water absorption ratio±SD* (%)	49.63±0.46	60.18±0.35	67.78±0.66	71.52±0.63	75.98±0.36
Weight Variation	(149-155mg) within the IP limits of ±7.5%				

* Average of three determinants

*In vitro* dissolution studies on the promising formulation (DCPC_4_), the control (DC_0_) and commercial conventional formulations (CCF) were carried out in pH 6.8 phosphate buffer, and the various dissolution parameter values viz., percent drug dissolved in 5 min, 10 min and 15 min (D_5_, D_10_ and D_15_), dissolution efficiency at 10 min (DE_10_ min)[12], t_50%_, t_70%_ and t_90%_ are shown in Table 3 and the dissolution profiles depicted in fig. 1. This data reveals that overall, the formulation DCPC_4_ has shown nearly two-and-half-fold faster drug release (t_50%_ 7.0 min) when compared to the commercial conventional tablet formulations of prochlorperazine maleate (t_50%_ 17.4 min) and released nearly three-times more drug than the control formulation in 10 min.

**TABLE 3:** *IN VITRO* DISSOLUTION PARAMETERS IN pH 6.8 PHOSPHATE BUFFER

Formulation code	D_5_ (%)	D_10_ (%)	D_15_ (%)	DE_10_ min (%)	t_50%_ (min)	t_70%_ (min)	t_90%_ (min)
DC_0_	10.00	18.00	20.00	26.28	>30	>30	>30
DCPC_4_	44.0	56.0	59.0	36.91	7.0	24.3	>30
CCF	24.00	32.00	44.00	24.75	17.40	>30	>30

DC_0_=Control formulation (without superdisintegrant), CCF=conventional commercial formulation, D_5_=percent drug released in 5 min, D_10_=percent drug released in 10 min, D_15_=percent drug released in 15 min, DE_10_ min=dissolution efficiency in 10 min, t_50%_=time for 50% drug dissolution, t_70%_=time for 70% drug dissolution, t_90%_=time for 90% drug dissolution.

**Fig. 1:**
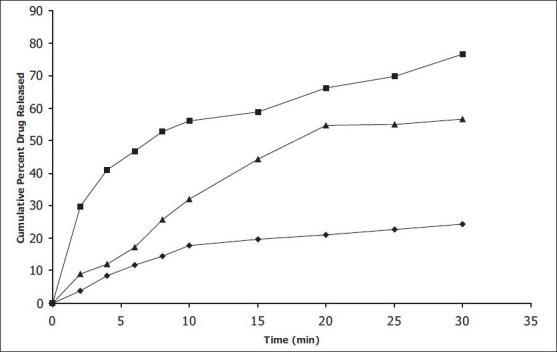
*In vitro* cumulative percent release versus time profile of promising formulations. Plot showing cumulative percent drug release in phosphate buffer (pH 6.8) from (–■–) combination of super-disintegrants used tablet, (–▲–) commercial conventional tablet and (–♦–) control formulation.

IR spectroscopic studies indicated that the drug is compatible with all the excipients. The IR spectrum of DCPC_4_ showed all the characteristic peaks of prochlorperazine maleate pure drug, thus confirming that no interaction of drug occurred with the components of the formulation. Short-term stability studies of the above formulation indicated that there are no significant changes in drug content and *in vitro* dispersion time at the end of 3 mo period (p<0.05).
